# Why should we not use APACHE II for performance measurement and benchmarking?

**DOI:** 10.5935/0103-507X.20170043

**Published:** 2017

**Authors:** Marcio Soares, Dave A. Dongelmans

**Affiliations:** 1 Department of Critical Care Medicine, Instituto D’Or de Pesquisa e Ensino, Rio de Janeiro (RJ), Brazil.; 2 DDepartment of Intensive Care Medicine, Academic Medical Center, University of Amsterdam - Amsterdam, The Netherlands.

The Acute Physiology and Chronic Health Evaluation (APACHE) is the most frequently used general severity-of-illness score in adult intensive care units (ICUs). APACHE scores use clinical, physiological and laboratory data observed at admission and during the first 24 hours after ICU admission. This is in order to estimate a given patient's severity of illness by providing a severity score and a probability of hospital death. Although severity of illness scores including APACHE scores should not be used to guide decisions for individual patients, they are useful for characterizing patients in clinical studies, evaluating ICU performance and benchmarking, for which case mix correction is needed.^(1)^

The first version of the APACHE score dates back to the early 80s.^(2)^ However, the APACHE I was too complex and time-consuming for routine use in the ICU. The APACHE II score was released more than 35 years ago in 1985 using data from 5,815 patients admitted between 1979 and 1982 to 13 hospitals in the United States (US).^(3)^ The number of variables was reduced from 34 to 12, and 50 admission disease groups were provided to improve the accuracy of outcome predictions. The APACHE II score was quickly adopted by ICUs worldwide and is the most used score in clinical studies to date. The APACHE III score was published in 1991 using data from 17,440 patients admitted to 40 US hospitals.^(4)^ More sophisticated statistical modeling approaches were used, and both the number of admission disease groups and the physiological variables were expanded.

Moreover, new equations to predict outcomes other than hospital mortality were provided. Updated versions of the APACHE III score were made available during the 90s. However, despite such updates, deteriorations in the model's performance over time indicated that the modeling of new equations would be required.^(5,6)^ Therefore, the APACHE IV, which represents the most recent version of APACHE scores, was introduced in 2006.^(5)^ Investigators used data from more the 110,000 ICU admissions in 45 hospitals that were still restricted to the US. The number of admission disease groups was expanded to 116.

Why are severity-of-illness scores regularly updated? It is not surprising that the performance of severity-of-illness scores deteriorates overtime. Such deterioration is invariably characterized by the worsening of discrimination (i.e., the ability to discriminate between survivors and non-survivors) and more importantly of models' calibration (i.e., the agreement between the observed and expected numbers of survivors and non-survivors across all the strata of probabilities of death), which can be ascribed to a series of reasons^(1,5)^ including advances in medical sciences; improvements in critical care treatment/management; improvements in patient management and therapeutic interventions; changes in case mix (e.g., aging populations, increased numbers of patients living with severe comorbidities) and changes in admission/discharge/end-of-life decision policies.

As survival from many conditions requiring critical care has improved over the last decades, prognostic scores tend to overestimate the probability of death as time passes, resulting in lower standardized mortality rates (SMR). The reporting of ICU quality and performance data is spreading quickly worldwide. In many countries, scoring systems have been used for benchmarking. However, validation studies are required before using these instruments in a specific country or region. Over the last decades, a series of studies using contemporary databases observed that the APACHE II is inaccurate for performance evaluation and benchmarking.^(7,8)^ As a consequence, several quality improvement initiatives replaced that score with updated versions (APACHE III and APACHE IV) or other severity-of-illness scores.^(7,9-13)^ Alternatively, some countries attempted to develop customized equations of the APACHE II scores to overcome the poor performance of the original equation.^(14)^ However, such practices were abandoned more than one decade ago due to both the availability of more recent versions of the APACHE and other scores and the decision to develop more locally adjusted instruments. A good example using both strategies is the Case Mix Programme of the Intensive Care National Audit & Research Center (ICNARC) in the United Kingdom (UK). During the mid 90s, customized versions of the APACHE II using UK-specific coefficients were made available, and recalibrations were regularly provided.^(9,14)^ In 2007, the ICNARC model was published, and it presented higher accuracy than other scores including the APACHE II and III scores and became the standard score for performance evaluation and benchmarking in the UK.^(9,14)^ A few years ago, the ICNARC made the decision to no longer recalibrate the APACHE II. The Dutch ICU registry (National Intensive Care Evaluation - NICE) receives data from 85 ICUs in the Netherlands.^(10)^ As of last year, they discontinued using the APACHE II in feedback reports due to a poor fit with the Dutch setting. Even after recalibration, the performance of the model was too low. In current feedback reports, the APACHE IV and the Simplified Acute Physiology Score (SAPS) II model are used. On the public website of the NICE, data on individual ICUs and aggregated data are shown using the APACHE IV model in various patient groups. An important advantage of the APACHE IV model is that it can also be used for cardiac surgery patients, enabling case mix-corrected benchmarking in this patient group. It must be said that due to the low occurrence of mortality in this patient group, it is difficult to find statistically significant differences between centers.

In summary, despite a myriad of arguments used by some clinicians and administrators (Table 1), the APACHE II score cannot be recommended for performance evaluation and benchmarking. For these purposes, updated versions of severity-of-illness scores that are appropriately validated to the country or region should be used.

**Table 1:** Frequently used arguments for continuing use of the Acute Physiology and Chronic Health Evaluation II score

– Familiarity with understanding the representativeness of a score for a given patient
– Familiarity with the use of the score
– Capacity to allow comparisons of SMRs over time
– Capacity to allow comparisons in terms of illness severity between new and old clinical trials
– As the score overestimates mortality, low SMRs can be easily demonstrated
– Simple inertia

SMR - standardized mortality rate.
